# Prediction of Gestational Diabetes by Measuring the Levels of Pregnancy Associated Plasma Protein-A (PAPP-A) During Gestation Weeks 11–14

**Published:** 2020

**Authors:** Somayeh Ramezani, Mahboubeh Ahmadi Doulabi, Hamid Saqhafi, Mahmood Alipoor

**Affiliations:** 1- Student Research Committee, School of Nursing and Midwifery, Shahid Beheshti University of Medical Sciences, Tehran, Iran; 2- Midwifery and Reproductive Health Research Center, Department of Midwifery and Reproductive Health, School of Nursing and Midwifery, Shahid Beheshti University of Medical Sciences, Tehran, Iran; 3- School of Paramedical Sciences, Qazvin University of Medical Sciences, Qazvin, Iran; 4- Department of Biostatistics, Faculty of Medicine, Qazvin University of Medical Sciences, Qazvin, Iran

**Keywords:** Gestational diabetes, Pregnancies, Pregnancy associated plasma protein A

## Abstract

**Background:**

The present study aimed to determine the association between pregnancy-associated plasma protein A (PAPP-A) and Gestational Diabetes Methods (GDM) to detect a risk factor for predicting GDM at gestational weeks 11–14.

**Methods:**

This analytical prospective study recruited 284 pregnant women presenting to six healthcare centers of Qazvin, Iran from February to December 2016. PAPP-A was measured at gestational weeks 11–14 and glucose tolerance test was conducted at gestational weeks 24–28. The participants were assigned into two groups of exposure (reduced PAPP-A) and non-exposure (normal PAPP-A). The association between GDM and PAPP-A was studied. The number of women in exposure group were 201 and 83 in the non-exposure group. Differences between groups were assessed by the Mann–Whitney, Chi-square, T test, logistic regression analysis and ROC Curve with a significance level of 0.05.

**Results:**

Twenty eight (33.73%) patients of the exposure group and 17 (8.46%) of non-exposure group developed GDM. There was a significant difference between the two groups in terms of GDM (p<0.001) and the risk of GDM was 3.98 fold higher in the exposure group (reduced PAPPA *mu/L*) than that of the non-exposure group (CI=2.39–6.65, p<0.001). Also, 53.3% of the exposure group and 46.7% of the nonexposure group were diagnosed with GDM (p=0.02). There was a significant difference in GDM between the groups and the risk of GDM was 1.85 times higher in the exposure group (reduced PAPPA MOM) than that in the control group (CI=1.09–3.15, p=0.020). According to the ROC curve results, PAPP-A and MOM are acceptable indicators for predicting GDM.

**Conclusion:**

A low PAPP-A level (MOM, MU/L) as a new risk factor for GDM can help early prediction and prevent maternal and fetal complication by timely treatment.

## Introduction

Diabetes is the most common medical condition in pregnancy. Gestational diabetes mellitus (GDM) is defined as various degrees of carbohydrate intolerance, initiated or diagnosed during pregnancy (1). This definition is used regardless of using insulin or not for treatment. The possible cause of this disorder is exacerbation of physiological changes in glucose metabolism (2). GDM occurs in 3–5% of pregnancies (3). Population-based studies have estimated that GDM affects about 200,000 pregnancies out of the 4 million births that occur annually in the United States (7%). The incidence of GDM has been doubled between 1994 and 2002, according to a population-based study. In 90% of women, the glucose intolerance is resolved following delivery. However, 15–60% of these women will have a risk of type 2 diabetes mellitus (T2DM) over the next 5 to 15 years (2–4). The prevalence of GDM has been reported at 4.8% in Iran (5).

Evidence suggests that GDM increases the risk of unfavorable fetal outcomes (6), leads to a large for gestational age (LGA) fetus, and increases the rate of cesarean section, fetal insulin level and infant’s obesity (7, 8). LGA and complications associated with its delivery, such as hard labor, dystocia and asphyxia, and respiratory distress are one of the most important causes of neonatal complications associated with GDM. GDM can lead to a decrease in baby’s blood sugar, cause seizure and jaundice, and delay in motor skills (2, 6, 9).

Mild hyperglycemia during pregnancy has adverse effects on maternal health, including increased prevalence of hypertension and cesarean delivery, preterm delivery, subsequent metabolic disturbances, and cardiovascular disease (2, 6). Also, a wide range of complications such as obesity and diabetes have been reported in their offsprings (2). GDM can lead to preeclampsia and fetal growth restriction (10).

Despite over 40 years of research, there is no general agreement on the best screening biomarker for GDM (2). Gestational diabetes diagnosis is currently performed with a 2 *hr* oral glucose tolerance test following consumption 75 *gr* of glucose at 24–28th weeks of gestation (11).

The pathophysiology of GDM takes place weeks to months before diagnosis and factors associated with this pathogenesis exist in blood before the clinical diagnosis of GDM (12). In GDM, the placenta endures changes such as increased hypervascularization, vascular dysfunction, chorionic villi, and dysfunction of blood stream of the placental villi. Several studies have reported the association between abnormal levels of placental proteins with GDM (12). It is reported that reduced number and diameter of villous capillaries lead to reduced pregnancy associated plasma protein A (PAPP-A) (14). PAPP-A is a zinc-binding matrix metalloproteinase produced by trophoblasts during pregnancy and can be identified from the 28th day of fertilization, resulting in increased level of insulin-like growth factor-1 (IGF-1) (14). Reduced blood level of PAPP-A leads to a decrease in IGF-1 (11–15). Lower levels of IGF lead to increased insulin levels, abnormal glucose clearance, and insulin resistance (15).

There was a significant association between low levels of PAPP-A and GDM, in some studies, so that PAPP-A levels in the first trimester of pregnancy were lower in the GDM group than in the control (12, 13, 15, 17, 25). While other studies did not show a significant relationship between PAPP-A in the first trimester of pregnancy and GDM requiring insulin therapy (18–20).

Considering the importance of GDM and its complications and tactful prevention, and controversial results of studies on PAPP-A and GDM (19, 20), in addition to the introduction of a new and available predicting biomarker for GDM, routinely tested during the 11–14th weeks of gestation, the aim of this study was to determine the relationship between PAPP-A levels and GDM in women who referred to health centers affiliated to Qazvin University of Medical Sciences, Iran.

## Methods

This prospective analytic study investigated 284 pregnant women at 11–14th weeks of gestation from February to December 2016.

For sampling and taking the socioeconomic level into account, Qazvin city was divided into three areas: north, center and south, and two health centers were randomly selected from each category. Then sampling continued in each center by purposive method to reach the calculated sample size.

Data collection tools included a demographic and obstetrics questionnaire, and a checklist to record PAPP-A, OGTT, as well as their device and determinant kits. Content validation was used to determine the validity of the demographic and obstetrics questionnaire. To determine PAPP-A levels, LaborDiagnostika Nord GmbH & CO.KG (LDN, Germany) kit was used. The immunoassay (Enzyme-Linked Immunosorbent Assay ELISA) was performed by full automatic ELISYS UNO Processor (Human Co. Germany). Pars Azmoon kits and the Alpha Classic Auto Analyzer (HITA-CHI 912) and SELECTRA E (Vitalab company Korea) were used to determine the blood glucose level. All PAPP-A tests were performed with one device and in one laboratory by ELISA method. Simultaneous observation was performed to test the expert’s reliability. For this purpose, 10 PAPP-A samples and 10 blood glucose samples were simultaneously examined by two individuals with the same educational level and the results were analyzed using Pearson correlation coefficient.

Pregnant women who referred to the selected health care centers in Qazvin were enrolled into the study, according to the inclusion criteria, including Iranian women aged 18–35, at 11–14th weeks of gestation based on LMP or first trimester’s ultrasonography, without history of GDM, type 1 and type 2 diabetes and family history of diabetes, no evidence of diabetes, no history of neonate with weight >4 kilograms, preeclampsia, eclampsia, hypertension, stillbirth, fetal abnormalities, frequent abortions, who did not smoke or substances abuse, who have not used assisted reproductive techniques (ARTs) in the current pregnancy, and had no history of known diseases (Cardiovascular disease, chronic hypertension, renal, hepatic, or hematopoietic disease, thyroid dysfunction, autoimmune disease, chronic inflammatory disease, hypersensitivity, polycystic ovary syndrome, metabolic syndrome and active infection at the time of sampling). Exclusion criteria consisted of unwillingness of the pregnant woman to continue the study, twin pregnancy, and inability to use glucose due to vomiting.

After receiving the introduction letter from the Qazvin University of Medical Sciences and obtaining written consent from the subjects, without imposing any costs on participants, sampler was housed in the centers and the first part of the checklist was completed, which comprised inclusion criteria, and if the patient had the conditions for entering the study, the second part of the checklist (Demographic characteristics and pregnancy records) was completed and then the pregnant women at 11–14th weeks of gestation were referred to the reference lab to carry out their routine screening tests of the first trimester. Blood samples were taken from the subjects in the reference laboratory and PAPP-A levels of all subjects were recorded by the researcher from the laboratory or the relevant checklist if the pregnant referred to the health center. Accordingly, subjects were categorized into two groups of exposure and non-exposure. Normal levels of PAPP-A was considered 943–1455 *MU/L* for 11–12th weeks of gestation, 1455–2243 for 12–13th weeks of gestation, and more than 2243 *MU/L* for 13th weeks of gestation. To report the results of PAPP-A in an international unit, Multiple of Median unit (MOM) was used and the results, obtained by *MU/L*, were corrected using Bentech software and MOM levels less than 0.4 were considered abnormal. The subjects were referred to the reference laboratory for follow up tests at the 24–28th weeks of gestation in order to carry out the routine GDM diagnosis test. Then, FBS and OGTT levels of the samples were recorded by the researcher from the laboratory or the relevant checklist if they referred to the health center. The assessment of glucose level was performed according to WHO 2013 guidelines. If FBS levels were ≥92, or 1 *hr* glucose was ≥180, or 2 *hr* glucose was ≥153, it was considered as GDM and mothers with GDM were referred to a gynecologist for treatment (10). The subjects were matched in both exposure and nonexposure groups in terms of maternal age, number of pregnancies, number of deliveries, and abortions. Data were analyzed using SPSS 21 software. The confidence interval coefficient was considered 95%. Descriptive statistics, t-test for quantitative variables, chi-square test for qualitative variables, Mann-Whitney test for ordinal variables, and logistic regression were used to determine relative risk to the relationship between GDM and PAPP-A levels and Area Under the Curve (AUC) were used for predicting gestational diabetes.

## Results

In this study, of 284 subjects in PAPP-A MU/L group, 201 were in the exposure group and 83 in the non-exposure group (Table 1). 10 pregnant women were excluded from the study due to inability to use glucose and vomiting after consuming and 3 were reluctant to continue the study, who were replaced with other subjects. The results of the study showed that the exposure and non-exposure groups were statistically similar in terms of confounding variables (Number of deliveries and abortions), and no significant difference was observed between the two groups (p=0.43 and 0.41, respectively). Confounding variables including body mass index (BMI), number of deliveries, and number of abortions and other obstetric characteristics are shown (Table 2). Statistical tests showed that the two groups were statistically similar in terms of age (p=0.99). There was a significant difference between the two groups in terms of BMI (p=0.003). Based on the results of the logistic regression, GDM had a statistically significant correlation with BMI and PAPP-A (both p=0.001). According to logistic regression results, by deleting the effect of BMI variable, the relative risk of GDM in patients with reduced PAPP-A levels was estimated 4.77 times than that of healthy people. The risk of gestational diabetes in individuals with abnormal BMI was 1.16 folds higher than those with normal BMI (Table 3). The statistical test showed a significant correlation between PAPP-A MOM and GDM (p=0.02) (Table 4).

**Table 1.** Distribution of pregnant women in two exposure and non-exposure groups according to their obstetrics characteristics

**Variables**	**Groups**		**Tests**	**p-value**

	**Exposure**	**Non-exposure**		
**Gravidity**	1.91	1.81	Mann–Whitney	0.76
**Parity**	0.66	0.74	Mann–Whitney	0.43
**BMI**	26.11	24.47	t-test	0.003
**Abortion**	0.23	0.17	Mann–Whitney	0.41

**Table 2.** Evaluation of gestational diabetes in two exposure and non-exposure groups (in MIU/L)

**PAPP-A**	**Groups**				**Chi-square p-value**	**RR 95%CI**

	**Gestational diabetes**		**Non-gestational diabetes**			

	**No.**	**Rate (%)**	**No.**	**Rate (%)**		
**Exposure (MU/L)**	28	33.73	55	66.26	0.001	3.98
**Non-exposure (MU/L)**	17	8.46	184	91.54		2.39–6.65
**Exposure (MOM)**	21	46.7	70	29.3	0.02	1.85
**Non-exposure (MOM)**	24	53.3	169	70.7		1.09–3.15

**Table 3.** Evaluation of gestational diabetes in two exposure and non-exposure groups (in MOM)

**PAPP-A (MU/L)**	**Groups**				**Chi-square p-value**	**RR 95%CI**

	**Gestational diabetes**		**Non-gestational diabetes**			

	**No.**	**Rate (%)**	**No.**	**Rate (%)**		
**Exposure**	21	46.7	70	29.3	0.02	1.85
**Non-exposure**	24	53.3	169	70.7		1.09–3.15

**Table 4.** Determination of the probability of gestational diabetes based on some risk factors

**Variables**	**Exp (B)**	**p-value**	**CI 95%**
**PAPP-A(MU/L)**	4.7	0.001	2.37–9.75
**BMI**	1.16	0.001	1.07–1.26

**Figure 1.**
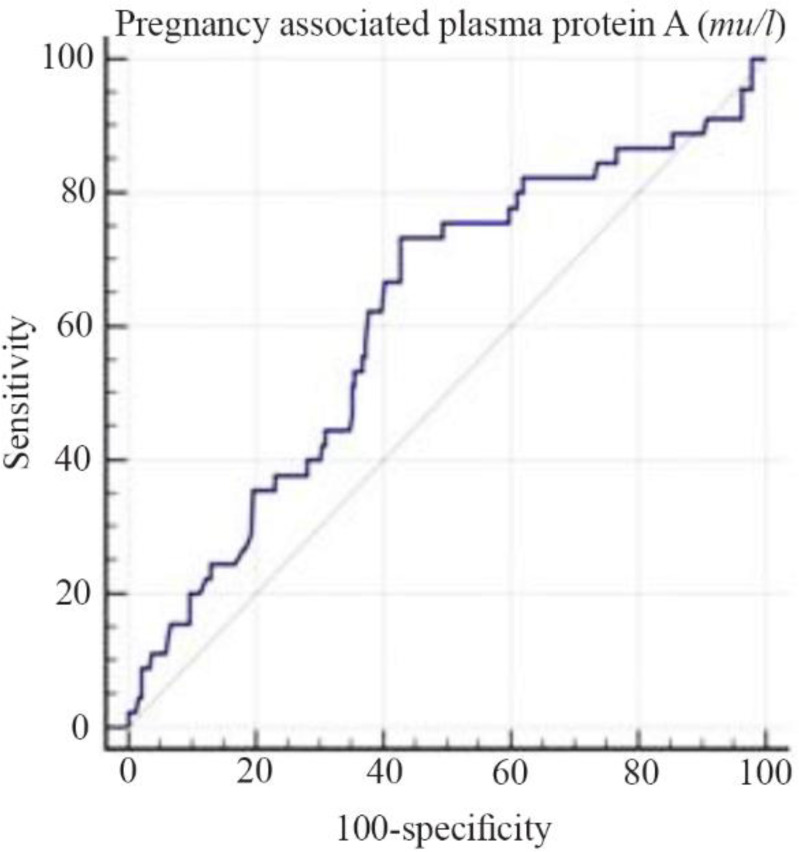
PAPP-A MU/L curve for prognosis of gestational diabetes in the study population

**Figure 2.**
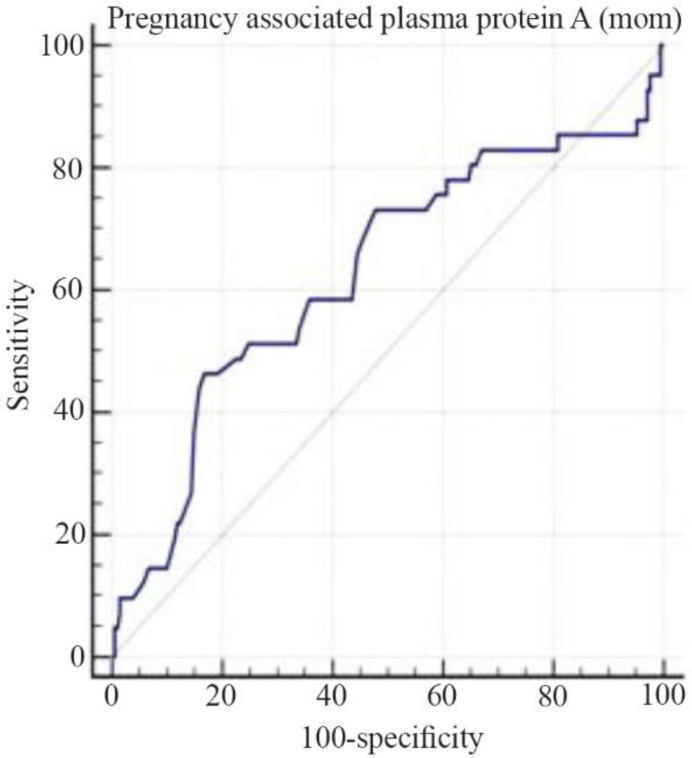
PAPP-A (MOM) curve for prognosis of gestational diabetes in the study population

The results of ROC curve showed the point 1896 as the best cut point for PAPP-A MU/L, with a maximum sensitivity of 73.33 and a maximum specificity of 57.32. Area Under the Curve (AUC) of 0.61% shows that PAPP-A MU/L is an acceptable index for predicting gestational diabetes (p=0.01) (Figure 1). Also, the point 0.32 was the best cut-off point for PAPP-A MOM, with a maximum sensitivity of 34.46 and maximum specificity of 83.25. The AUC is 0.62% which indicates that PAPP-A MOM is an acceptable index for predicting GDM (p=0.017) (Figure 2).

## Discussion

The present study confirmed the association between low PAPP-A and GDM. Other studies have also stated the relationship between low PAPP-A with adverse pregnancy outcomes (12). In the study of Jelliffe et al. (2015), there was a correlation between low PAPP-A and pre-eclampsia (21). In the study of Giudice et al. (2015), there was a relationship between low PAPP-A and low birth weight (LBW) (16). In the study by Spencer et al. (2008), there was a relationship between the low PAPP-A levels and small for gestational age (SGA) and preterm delivery (21, 23). In the present study, the relationship between low PAPP-A and GDM was studied.

Based on Bennenti et al. (2014), a significant association was found between low PAPP-A levels and GDM (13). In another study, Beneventi et al. (2011) reported significantly lower PAPP-A levels in the first trimester in subjects with GDM than in the control group, as well as an inverse association between BMI and low PAPP-A that could be a confounding variable in expressing PAPP-A (12). According to Inan et al., Sweeting et al., Donavan et al., and Shah k et al., a significant association between low PAPP-A levels and GDM exists (10, 26, 27, 28, 29, 30).

In the present study, the effect of this confounding factor was modified by statistical tests. The limitations mentioned in the study by Beneventi et al. (2011) was the type of study (Case-based) and lack of using a new criterion for diagnosis of GDM (12), while the present study is a prospective analytical study using new criteria for diagnosis of GDM. In study by Ong et al. (2000), low level of PAPP-A was associated with pre-diabetic state and GDM and PAPP-A was 20% lower than that of the control group. In this study, there was a statistically significant relationship between low PAPP-A le vels and eclampsia, IUGR, and abortion (24). In the study of Lovati et al. (2013), low PAPP-A MOM and MU/L levels in the first trimester of pregnancy were associated with a high risk of insulin therapy in diabetic pregnant women (15). In the study of Ledesma et al. (2014), in women over 70 *kg*, weight lower PAPP-A was associated with higher risk of GDM (17). On the other hand, the results of Husslein et al. (2012) did not show any difference between the amount of PAPP-A MOM in the first trimester and GDM (19). The reason for this lack of consistency can be the difference between the guidelines used for the diagnosis of GDM and the use of the White’s classification for inclusion of participants into the study, as well as the number of samples studied. Also, in the study of Husslein et al., the relationship between PAPP-A and insulin-dependent GDM was studied, while in the present study, the relationship between PAPP-A and GDM has been investigated regardless of the need for insulin therapy or dietary regimen. On the other hand, Husslein’s study did not control the variables including age and number of abortion and deliveries, while Cunningham et al. (2014) identified age and number of abortion and deliveries as the risk factor of GDM (2); in the present study, the effect of these confounding factors has been modified. Furthermore, Spencer et al. (2005) found no significant relationship between insulin-dependent diabetes and PAPP-A levels, human chorionic gonadotropin (HCG) and NT (Nuchal translucency) (20). The results of this study are not in line with the present study. The causes of discrepancy of our results with that of Spencer can be lack of controlling factors such as age, pregnancy history and medical history of diseases and that the case group in Spencer’s study included women with GDM requiring insulin therapy, while in the present study, all confounding factors were controlled and the association between PAPP-A and GDM has been studied with or without insulin therapy. Also in the study of Chenhong et al. and Maymon et al., no significant association between low PAPP-A levels and GDM was found (31, 32).

PAPP-A is a zinc-binding matrix metalloproteinase produced by trophoblasts during pregnancy and in corpus luteum and granoloza in nonpregnant women. In laboratory models, PAPP-A expression increases in damaged vessels and human plug atherosclerosis by stimulation of Tumor Necrosis Factor (TNF) and Interleukin-1 beta (12). In patients with cardiovascular disease, normal PAPP-A compared with high levels predicts reduced risk of death and heart failure. The relationship between macrosomia and GDM suggests the role of PAPP-A as part of IGF control system in trophoblast as an insulin-like growth factor-binding protein (IGFBP-4). Reduced PAPP-A leads to a decrease in IGF and an increase in glucose and amino acids produced by trophoblast (25). One of the possible mechanisms for explaining the association between abnormal PAPP-A and GDM is the decreased IGF, which leads to increased insulin, glucose clearance, and insulin resistance (15). In the study of Pellitero et al. (2008), the relationship between PAPP-A and glucose control was studied in non-pregnant diabetic patients. In this study, there was an inverse relationship between hemoglobin A1C and PAPP-A in diabetic patients, reflecting the effect of glucose control on PAPP-A expression (33).

Savvidou et al. (2010) investigated several powerful predictors of GDM, including demographic information and new biochemical markers such as lipid, C-reactive protein, Gamma Glutamyl transferase, adiponectin, E-Selectin, and tissue plasminogen activator in the first trimester of pregnancy. The results of this study showed that demographic data including age and BMI, history of GDM and family history of diabetes, smoking, and race, obstetric characteristics including number of births, and biochemical markers, including reduced HDL (high Density Lipoprotein) and tissue plasminogen activator had a significant relationship with GDM (33). Nanda et al. (2011) used demographic information and laboratory markers, including adiponectin, Follistatin like 3, and Sex Hormone Binding Globulin (SHBG) for early prediction of GDM. The results of this study showed that demographic data, decreased adiponectin, and SHBG had a significant relationship with the onset of GDM (35). Teed et al. (2011) introduced the history of GDM as the strongest predictor of GDM for intervention in the first trimester (36).

The strengths of this study included the prospective design and investigation of all factors that develop GDM and control these factors, as well as using the newest diagnostic criteria for GDM. Reduced PAPP-A is also associated with preeclampsia, eclampsia, IUGR, and preterm labor (20–22). The consequences of reduced PAPP-A increased the probability of referral of exposed pregnant women to healthcare medical centers than that of non-exposed pregnant mothers. As a result, this prospective study also reduced the bias of choice. In this study, due to the fact that PAPP-A is currently used in Iran’s health care medical centers for routine screening of fetal abnormalities, it is preferable to other studies in research centers requiring additional testing that would not be economically beneficial to pregnant women. GDM has a relatively high prevalence. Identification of a new risk factor for GDM helps early prediction of at-risk pregnant women and by preventive measures including diet and exercise counseling and advice on proper weight gain and timely treatment, the maternal and fetal complications could be prevented. The main limitation of this research is recording the variables, affecting the independent variable, based on the patients’ statements that would include memory bias of not considering the factors and medical conditions affecting PAPP-A levels. Conducting clinical and laboratory tests for diagnosis of medical conditions and possible factors affecting PAPP-A levels requires great time and costs, which was not in accordance with the financial conditions of the research team.

## Conclusion

Considering the low levels of PAPP-A MU/L and MOM as a new risk factor for GDM can help early prediction of at-risk women, and as far as this test is requested routinely at 11–14th weeks of gestation in health centers for pregnant women, all health care providers should receive the necessary training in this regard and in case of low PAPP-A levels, they should diagnose at-risk patients and prevent maternal and fetal complications by tactful and timely treatment with less time and costs.
